# Docking Studies in Target Proteins Involved in Antibacterial Action Mechanisms: Extending the Knowledge on Standard Antibiotics to Antimicrobial Mushroom Compounds

**DOI:** 10.3390/molecules19021672

**Published:** 2014-01-29

**Authors:** Maria José Alves, Hugo J. C. Froufe, Ana F. T. Costa, Anabela F. Santos, Liliana G. Oliveira, Sara R. M. Osório, Rui M. V. Abreu, Manuela Pintado, Isabel C. F. R. Ferreira

**Affiliations:** 1 Centro de Investigação de Montanha (CIMO), ESA, Instituto Politécnico de Bragança, Campus de Santa Apolónia, Apartado 1172, Bragança 5301-855, Portugal; E-Mails: maria.alves@ipb.pt (M.J.A.); hfroufe@ipb.pt (H.J.C.F.); ruiabreu@ipb.pt (R.M.V.A.); 2 CBQF-Escola Superior de Biotecnologia — Universidade Católica Portuguesa Porto, Rua Dr. António Bernardino de Almeida, Porto 4200-072, Portugal; 3 Centro Hospitalar de Trás-os-Montes e Alto Douro- Unidade de Chaves, Av. Dr. Francisco Sá Carneiro, Chaves 5400-249, Portugal; 4 ESSA, Instituto Politécnico de Bragança, Campus de Santa Apolónia, Apartado 1172, Bragança 5301-855, Portugal; E-Mails: filipanaregua25@gmail.com (A.F.T.C.); anabela_santos99@hotmail.com (A.F.S.); a24859@alunos.ipb.pt (L.G.O.); s.rafaela@hotmail.com (S.R.M.O.)

**Keywords:** mushrooms, antimicrobial compounds, antibiotics, target proteins, docking studies

## Abstract

In the present work, the knowledge on target proteins of standard antibiotics was extended to antimicrobial mushroom compounds. Docking studies were performed for 34 compounds in order to evaluate their affinity to bacterial proteins that are known targets for some antibiotics with different mechanism of action: inhibitors of cell wall synthesis, inhibitors of protein synthesis, inhibitors of nucleic acids synthesis and antimetabolites. After validation of the molecular docking approach, virtual screening of all the compounds was performed against penicillin binding protein 1a (PBP1a), alanine racemase (Alr), d-alanyl-d-alanine synthetase (Ddl), isoleucyl-tRNA sinthetase (IARS), DNA gyrase subunit B, topoisomerase IV (TopoIV), dihydropteroate synthetase (DHPS) and dihydrofolate reductase (DHFR) using AutoDock4. Overall, it seems that for the selected mushroom compounds (namely, enokipodins, ganomycins and austrocortiluteins) the main mechanism of the action is the inhibition of cell wall synthesis, being Alr and Ddl probable protein targets.

## 1. Introduction

The classification of antibiotics is based on their mechanism of action, and the main groups include inhibitors of cell wall synthesis, inhibitors of protein synthesis, inhibitors of nucleic acids synthesis and antimetabolites [1]. In general antibiotics inhibit these routes by interacting with specific cell proteins, usually responsible for defined activity.

Antimicrobials acting at the cell wall level are the most selective, being bactericidal and presenting a high therapeutic index, since inhibition of peptidoglycan synthesis leads to cell lysis [2]. There is a large diversity of antibiotics that can act in different phases of peptidoglycan biosynthesis, namely in the cytoplasmic, membrane and parietal phases [3]. β-Lactams act entirely outside the cell membrane, in the final (parietal) phase of peptidoglycan biosynthesis [3,4]. They act in penicillin binding proteins (PBPs), which are responsible for transpeptidation, transglucosylation and carboxypeptidation reactions. These antibiotics have β-lactam rings with spatial structures similar to that of the acyl-d-alanyl-d-alanine residues in peptidoglycan chains (natural ligand of PBPs) that link with and inhibit those proteins [3]. Fosfomycin acts in the membrane phase and prevents the transference of enolpyruvate to the intermediate uridine diphosphate-*N*-acetylglucosamine (UDP-NAG), compromising the formation of UDP-NAG-enolpyruvate that would be reduced for the synthesis of UDP-N-acetylmuramic acid (UDP-NAMA). d-Cycloserine acts in the cytoplasmic phase and it is a competitive inhibitor of alanine racemase (Alr) and d-alanyl-d-alanine synthetase (Ddl), inhibiting the incorporation of d-alanyl-d-alanine in UDP-NAMA tripeptide [3,4]. Nevertheless, other antibiotics such as glycopeptides (e.g., vancomycin) and bacitracin act in the membrane phase [3]. Bacitracin links to the lipid carrier, undecaprenyl-pyrophosphate (lipid-P-P), preventing dephosphorylation by membrane phosphatase [3,4]. On the other hand, glycopeptides bind to the d-alanyl-d-alanine moiety in the precursor of the peptidoglycan (NAG-NAMA-pentapeptide-P-P-lipid), in the interface between cytoplasmic membrane and cell wall, thus preventing the transfer of newly synthesized drivers to the array parietal growth [2].

Some antibiotics acting in protein synthesis affect bacteria ribosomes, namely 30S and/or 50S subunits, inhibiting the protein synthesis at the initial or elongation phases, or inducing the synthesis of abnormal proteins [5]. Macrolides, chloramphenicol, lincosamides, streptogramins and oxazolidones act at the 50S subunit, while aminoglycosides, spectinomycin and tetracyclines exert their antibacterial action in the 30S subunit [3]. Chloramphenicol exhibits a broad bacteriostatic spectrum [5]; it blocks the principal ribosome functions, including peptidyl transferase activity and aminoacyl-tRNA elongation, inhibiting peptidyl transferase by interfering in tRNA positioning [6,7]. Mupirocin is a bacteriostatic antibiotic with higher activity upon Gram-positive bacteria by inhibiting isoleucyl-tRNA synthetase (IARS), preventing the incorporation of amino acid isoleucine in peptides [3].

Quinolones interfere in DNA replication by inhibiting the activity of bacteria type II topoisomerase (DNA gyrase) and type IV topoisomerase [8]. Rifampicin inhibits RNA polymerase activity by linking to β-subunit, preventing the synthesis of mRNA [2].

Folic acid is essential for the synthesis of nitrogen base purines and pyrimidines and, consequently, for the synthesis of DNA. Antimetabolites act synergistically in two different points of folic acid formation, exhibiting bacteriostatic activity. Sulfonamides, dapsone and *p*-aminosalicylic acid are some examples [2,9]. Sulfonamides inhibit the action of dihydropteroate synthetase (with *p*-aminobenzoic acid (PABA) as substrate), preventing the synthesis of dihydrofolic acid. Trimetoprim blocks enzymatic activity of dihydrofolate reductase (DHFR), which is responsible for the formation of tetrahydrofolic acid [9].

**Figure 1:**
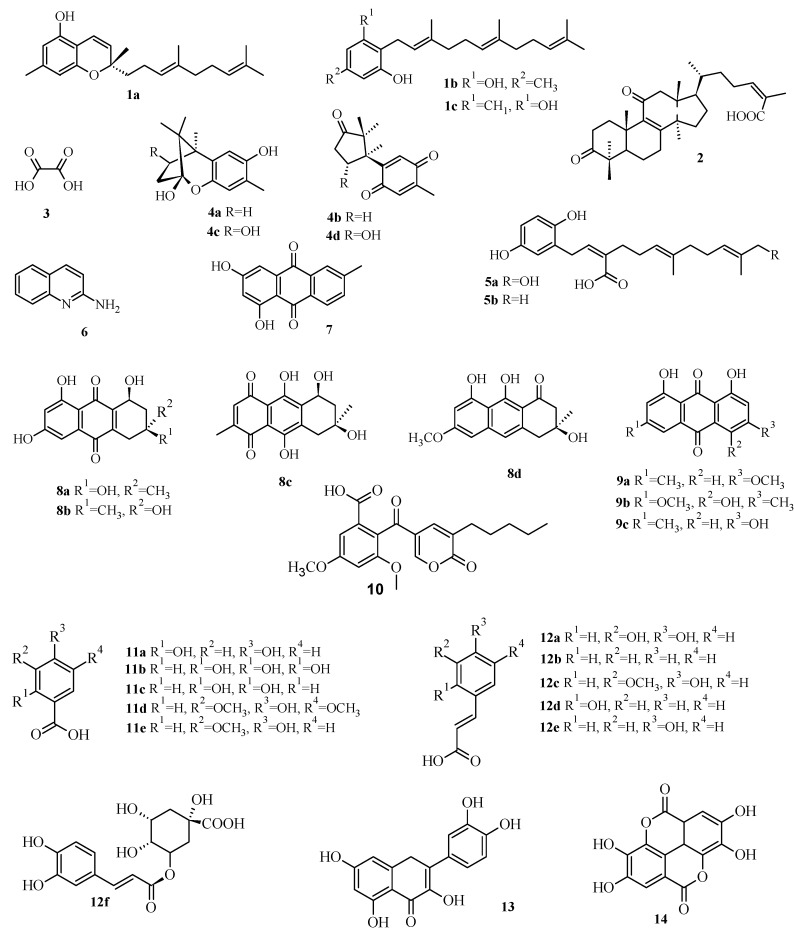
Chemical structures of the mushroom compounds pointed out as antimicrobial agents.

Considering bacterial evolution and the current increase of antibiotic resistance, the discovery of new natural compounds that can be used to treat infections with lower secondary effects than existing antibiotics is becoming crucial, in order to guarantee the health of future generations [10]. In this regard, mushrooms have proved to be particularly interesting sources of antimicrobial compounds [11,12] although their mechanisms of action are not yet fully described. Herein, we intended to extend the knowledge on target proteins of standard antibiotics to antimicrobial mushroom compounds, in order to predict possible interactions between the natural compounds and target proteins that would allow understanding and describing the mechanism of action. Therefore, docking studies were performed for 34 antimicrobial compounds (Figure 1) in order to evaluate their affinity to bacterial proteins that are known targets for some antibiotics.

## 2. Results and Discussion

### 2.1. Protein Targets

A docking study of target proteins involved in antibacterial mechanisms was performed to extend the knowledge on standard antibiotics to mushroom compounds with reported antibacterial activity. The proteins used were the following: PBP1a from *Acinetobacter baumannii*, Alr from *Escherichia coli*, IARS and Ddl from *Thermus thermophilus*, DNA gyrase subunit B and DHFR from *Staphylococcus aureus*, and TopoIV and DHPS from *Streptococcus pneumonia*.

The protein structures used in this work for IARS and Ddl were from *Thermus thermophilus*, although the best targets would be from *S. aureus* or *Mycobacterium tuberculosis* (also available in PDB). However, it was not possible to validate the docking methodology for these protein structures, as the obtained docking pose for the ligand didn’t superimpose well with the co-crystallized ligand structure. In fact they present RMSD values well above the 2 Å value that is usually considered a good threshold value for validating a structure for use in molecular docking. *M. tuberculosis* is a significant cause of morbidity and mortality in undeveloped countries, but due to its easy dissemination among patients with HIV and immunosuppression, it is also starting to be considered a serious public health problem in developed countries [2]. Regarding *S. aureus*, despite the presence in the human flora of skin and especially nasal mucosa, it is an opportunistic pathogen frequently found in nosocomial infections (but also in community infections, especially related with skin conditions) that may lead to severe infections, including septicemias [2]. Regarding DNA gyrase, the crystal structure used presented the co-crystallized ligand 07N bound to the ATP binding site, so for DNA gyrase we are analysing the potential of the studied compounds to bind to the ATP binding site. For TopoIV the co-crystallized ligand LFX was bound to DNA binding site and in this case we are analysing the potential of the studied compounds to bind to the DNA binding site. It should be highlighted that the docking simulation in this study was performed between a rigid receptor (protein) and a flexible ligand (compound). Thus, possible target proteins with high flexibility in their active sites were discarded.

### 2.2. Docking and Scoring Validation

In order to validate the docking approach for the protein structures used, the respective co-crystallized ligands were docked to the active site of each protein using AutoDock4. Some protein structures presented natural substrates as a co-crystallized ligand whereas in others the co-crystallized ligand was a known inhibitor (Table 1), in both cases the same docking and scoring validation process was used. Each co-crystalized ligand, was previously removed from the respective protein binding site. The predicted docking pose was compared with the experimental co-crystallized binding pose. This docking validation was not performed for the Alr structure where the co-crystallized ligand is a covalently linked prosthetic group (pyridoxal-5′-phosphate), as AutoDock4 is not able to handle covalent bonds.

**Table 1:** Values of Ki predicted by AutoDock4, Xscores and experimental values of Ki.

Protein	Class	Ligand	PDB (ID)	RMSD (Å)	Predicted AutoDock4 Ki (µM)	Predicted Xscore Ki (µM)	Experimental Ki (µM)
PBP1a	A	PNM ^b^	3UDI	1.33	5.49	1.862	-
Ddl	A	ATP ^a^	2ZDQ	1.49	0.006	0.478	-
IARS	B	ILA ^b^	1JZQ	1.13	0.203	0.707	0.006
DNA Gyrase	C	07N ^b^	3TTZ	0.23	0.419	0.457	0.004
TopoIV	C	LFX ^b^	3RAE	1.66	0.139	0.245	-
DHPS	D	PMM ^a^	2VEG	0.57	4.382	7.413	33
DHFR	D	Q2 ^b^	3SRW	1.85	0.050	0.089	0.00003
				r	0.90	0.99	style="border-top: solid thin"
				ρ	0.80	1	

A—Inhibitors of cell wall synthesis; B—Inhibitors of protein synthesis; C—Inhibitors of nucleic acid synthesis; D—Antimetabolites. a—Natural Substrate; b—Inhibitor.

All the predicted docking poses presented a root mean square deviation (RMSD) lower than 2 Å, when compared to the experimental co-crystallized binding pose (Table 1). This is a strong evidence that AutoDock4 can predict docking poses accurately, as 2 Å is usually considered a good threshold value for RMSD.

In order to validate scoring predictability, Ki values estimated by AutoDock4 or Xscore were compared with experimental Ki values, when available, by calculating ρ and r (Table 1); r measures the correlation between predicted pKi by AutoDock4 or Xscore and experimental pKi; ρ measures the rank correlation between predicted Ki by AutoDock4 or Xscore and experimental Ki.

Xscore allowed slightly better results (ρ = 1 and r = 0.99) than AutoDock4 (ρ = 0.80 and r = 0.90). Xscore correctly ranked all the 4 compounds tested (ρ = 1), with a higher linear correlation between the predicted and experimental results (r = 0.99). Therefore, Xscore may be a better scoring function for the protein structures studied when compared to AutoDock4.

### 2.3. Virtual Screening of Antimicrobial Mushroom Compounds

After validation of the molecular docking approach and scoring predictability with ligands (positive controls), virtual screening of the 34 compounds dataset was performed against PBP1a, Alr, Ddl, IARS, DNA gyrase subunit B, TopoIV, DHPS and DHFR using AutoDock4. The 34 compounds (Figure 1) used as dataset were previously identified in mushrooms and were previously pointed out by our research group as being antimicrobial agents [11,12].

All top docking structures predicted by AutoDock4 were rescored using Xscore, and further manually analyzed. The compounds out of the active site were presented in Table 2 in bold. Xscore scoring was also normalized for an easier interpretation, being divided in three parts: low predicted activity comprises compounds that scored above 10 μM (Table 2 red); medium predicted activity includes compounds that scored between 10 and 1 μM (Table 2 yellow); high predicted activity encloses compounds that scored below 1 μM (Table 2 green). Five proteins (PBP1a, Alr, Ddl, TopoIV and DHFR) had compounds with high predicted activity (Table 2).

**Table 2:** Predicted Xscore Ki (µM) of mushroom compounds with antimicrobial activity against proteins involved in antimicrobial mechanisms of action.

Compound	Code	PBP1a	Alr	Ddl	IARS	DNA Gyrase	TopoIV	DHPS	DHFR
Confluentin	**1a**	10.72	0.17	1.45	*162.18*	8.56	**0.26**	6.46	**0.44**
Grifolin	**1b**	1.91	1.35	0.1	251.19	28.18	0.83	4.47	1.75
Neogrifolin	**1c**	0.91	*1.12*	0.09	45.71	37.15	1.07	**1.51**	2.33
3,11-Dioxolanosta-8,24(Z)-diene-26-oic acid	**2**	**0.07**	*14.45*	**0.01**	**3.16**	**6.31**	*0.23*	4.27	*0.15*
Oxalic acid	**3**	112.2	*134.9*	*87.1*	*354.81*	*89.13*	77.62	158.49	*124.9*
Enokipodins A	**4a**	1.23	0.36	0.15	50.12	9.55	*2.75*	*63.1*	3.55
Enokipodins B	**4b**	1.62	0.51	4.57	23.44	21.38	*7.76*	*131.8*	5.62
Enokipodins C	**4c**	1.02	0.25	0.19	60.26	10.23	*6.03*	*70.79*	4.04
Enokipodins D	**4d**	1.17	0.52	0.34	218.78	32.36	*7.59*	2.4	4.68
Ganomycin A	**5a**	1.78	0.49	0.33	457.09	69.18	1.62	14.45	0.86
Ganomycin B	**5b**	1.66	**0.15**	0.3	40.74	48.98	0.66	5.37	0.46
2-Aminoquinoline	**6**	*12.59*	5.37	2.4	*4.47*	54.95	25.12	25.12	5.71
6-Methylxanthopurpurin-3- *O*-methyl	**7**	2.95	0.66	0.38	28.84	30.9	0.93	3.09	1.25
Austrocortilutein A	**8a**	3.24	0.3	0.4	12.88	14.13	*11.75*	2.14	5.37
Austrocortilutein B	**8b**	2.51	0.3	0.35	13.18	19.5	0.54	2.14	5.5
Austrocortirubin	**8c**	2.88	0.4	0.32	41.69	36.31	*22.39*	21.38	*25.51*
Torosachrysone	**8d**	2.04	0.26	0.32	58.88	16.98	10.23	*2.95*	4.33
Physcion	**9a**	1.91	0.6	0.3	30.2	7.08	0.72	*3.39*	1.62
Erythroglaucin	**9b**	2.69	0.55	0.19	26.3	28.84	0.62	2.63	2.15
Emodin	**9c**	2.57	0.41	0.32	40.74	11.75	0.72	2.57	1.12
Coloratin A	**10**	1.12	2	0.25	61.66	16.98	0.54	1.58	0.91
2,4-Dihydroxybenzoic acid	**11a**	16.22	7.94	9.55	*7.76*	*2.34*	9.55	16.98	15.37
Gallic acid	**11b**	19.05	8.32	17.38	*6.76*	*35.48*	8.91	87.1	19.65
Protocatechuic acid	**11c**	18.2	6.17	15.85	*8.13*	33.88	10.96	*17.78*	17.65
Syringic acid	**11d**	18.2	10	14.13	316.23	*74.13*	9.55	*15.14*	25.31
Vanillic acid	**11e**	19.05	9.77	13.8	*7.94*	34.67	10.47	*18.62*	22.91
Caffeic acid	**12a**	7.94	3.8	5.37	114.82	46.77	5.89	*195*	*33.11*
Cinammic	**12b**	10.23	4.27	5.01	*3.63*	46.77	8.71	12.02	*18.48*
Ferulic acid	**12c**	12.88	4.37	5.89	*269.15*	20.89	6.03	9.55	*36.59*
*o*-Coumaric acid	**12d**	12.3	4.17	4.07	*204.17*	22.91	6.46	30.2	11.66
*p*-Coumaric acid	**12e**	10.72	4.9	6.03	154.88	16.98	6.46	40.74	*19.8*
Chlorogenic acid	**12f**	1.55	*38.02*	0.21	*316.23*	*23.99*	*7.59*	42.66	3.6
Quercetin	**13**	1.74	0.52	0.35	97.72	19.5	0.52	13.8	2.53
Ellagic acid	**14**	4.27	0.56	0.58	58.88	46.77	4.68	2.63	8.45

Scores below 1 µM were presented at green, scores between 10 and 1 µM were presented at yellow and scores above 10 µM were p resented at red. Compounds with the best score for each protein was highlighted in bold. Compounds docked out of the active site were presented in italics.

Still, it is important to point out that, apart from DHFR, the protein used for the validation step (IARS, DHPS and DNAg) presented lower predicted scores. Although this circumstance is probably just a coincidence, it highlights that fact that interpreting docking predicted scores should always the taken with caution.

The target proteins, which are involved in the cell wall synthesis (PBP1a, Alr and Ddl), had compounds scored lower than 1 μM. Regarding PBP1a, the best results were obtained with neogrifoline and 3,11-dioxolanosta-8,24(Z)-diene-26-oic acid; the latter also presented the highest score for Ddl. Otherwise, the score of the mentioned compound for Alr was low, being the highest score obtained with ganomycin B.

Considering the lowest predicted Ki of some compounds for Alr and Ddl, namely the enokipodins, ganomycins and austrocortiluteins, these proteins may be involved as possible targets of such antimicrobial compounds. On the other hand, IARS revealed the highest scores for most of the 34 compounds tested, suggesting that IARS is not a good target for the tested compounds, specially taking into account that ILA, the co-crystallyzed ligand inhibitor, presents an experimental Ki value of 0.006 µM. Otherwise, other mechanism of protein synthesis inhibition cannot be excluded. The protein targets involved in nucleic acid synthesis (DNA gyrase and TopoIV) have two active sites. Herein, ATP binding site was tested in DNA gyrase, while DNA binding site was tested in TopoIV. The results revealed a higher affinity of the compounds towards the DNA binding site, which is also a target for antibiotic quinolones that share some features with the best scored compounds (e.g., confluentin, grifolin and ganomycin B). It is important to point out that the best scores for DNA gyrase were all above 1 µM, a value that fares weakly with the co-crystallyzed ligand inhibitor 07N that presents a very potent experimental Ki of 0.004 µM.

In regard to the protein targets of antimetabolites (DHPS and DHFR), in general DHFR presented better scores than DHPS. However it is important to note that, for DHFR, the co-crystallized ligand was the inhibitor Q7 with an experimental Ki of 0.00003 µM, a far more potent Ki than the best predicted Ki of 0,15 µM for 3,11-dioxolanosta-8,24(Z)-diene-26-oic acid. On the other hand, altough the best predicted Ki for DHPS was a less potent 1.51 µM value for neogrifolin, it is still more potent than the co-crystallyzed natural substrate PMM that presents a higher experimental Ki value of 33 µM.

Phenolic acids, compounds **11** and **12**, gave high predicted Ki values in all the tested proteins, suggesting that these targets are not related to their mechanism of action. However, it is important to highlight the affinity of chlorogenic acid for Ddl.

**Figure 2:**
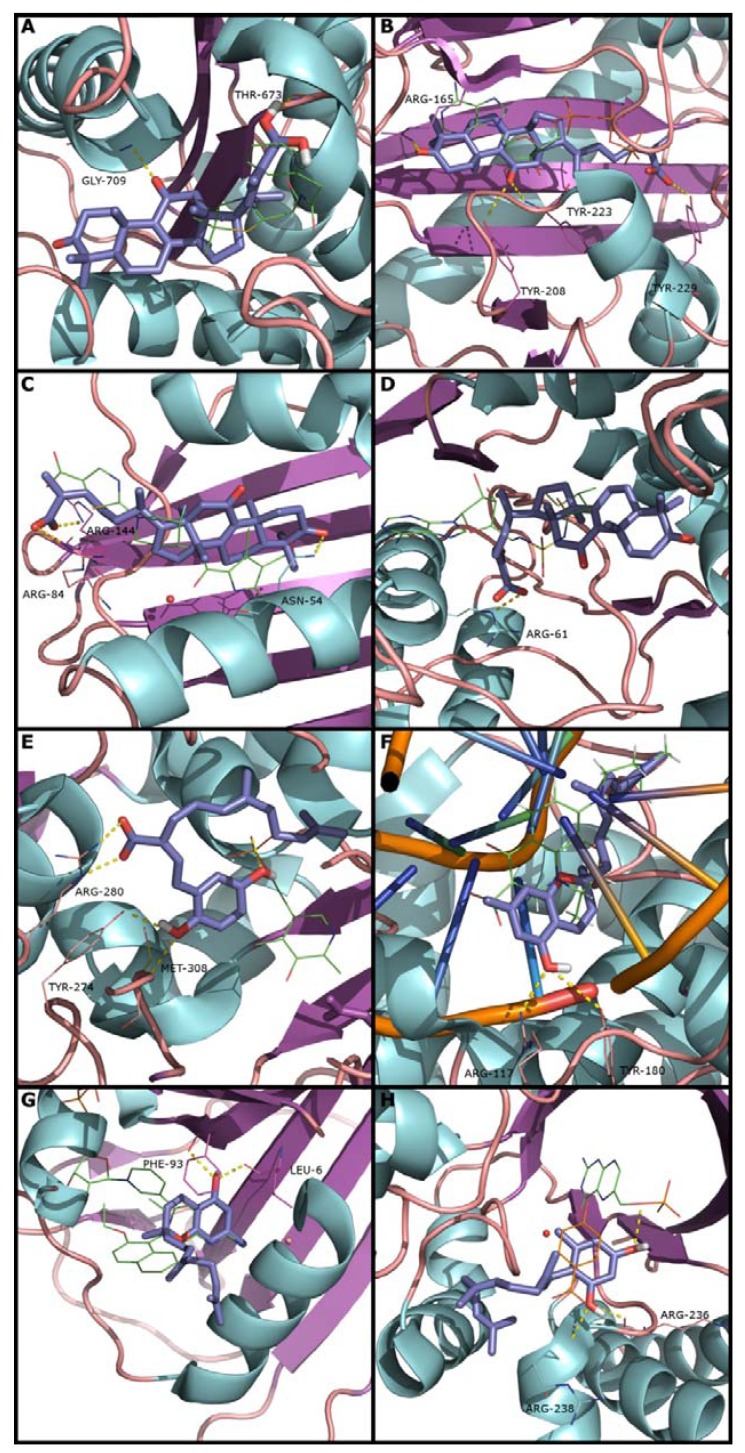
Docking pose: PBP1a (**A**), Ddl (**B**), DNA Gyrase (**C)** and IARS (**D**) with 3,11-dioxolanosta-8,24(Z)-diene-26-oic acid; Alr (**E**) with ganomycin B; TopoIV (**F**) and DHFR (**G**) with confluentin; DHPS with neogrifolin (**H**). All the proteins are presented in cartoons, the predicted poses are presented in purple sticks and co-crystallized ligands presented in green lines. In 2H, p-hydroxybenzoic acid (orange line), which is a ligand co-crystallized with the other DHPS structure (3TYB), is superimposed.

3,11-Dioxolanosta-8,24(Z)-diene-26-oic acid (2) was the best scored compound for PBP1a, with a predicted Ki of 0.07 μM (Table 2). This compound interacts with PBP1a by forming hydrogen bonds with GLY-709 and THR-673 (Figure 2A) explaining the high affinity for this protein, and suggesting a possible mechanism of the action. Compound 2 also showed the best score for Ddl, DNA gyrase and IARS (Table 2), all of which are ATP dependent proteins. As it can be observed in Figure 2B–D, AutoDock4 predicted a pose of compound 2 that occupies the same area of ATP in each of the three proteins. Figure 2C presents the binding pose of compound 2 against Ddl, indicating that the compound interacts with Ddl by forming hydrogen bonds with TYR-218 and TYR-229. Compound 2 interacts with DNA gyrase by forming three hydrogen bonds with ASN-54, ARG-84 and ARG 144 (Figure 2E). IARS interacts with compound 2 by forming one hydrogen bond with ARG-61 (Figure 2D). Nevertheless, the best score of compound 2 was against Ddl (0.01 μM), corresponding also to the best score among all the results. Scores of compound 2 against DNA gyrase and IARS indicate that this compound has a much lower affinity for those proteins, excluding these proteins as main target for this compound.

The best scored compound for Alr was ganomycin B (**5b**) with a predicted score of 0.15 μM. Figure 2E shows the binding pose predicted by AutoDock4 for ganomycin B against Alr. This compound interacts by forming hydrogen bonds with residues ARG-280, TYR-274 and the prosthetic group pyridoxal-5′-phosphate presented in the active site. Furthermore this protein has a very narrow access to the active site, which becomes the main difficulty for predicting if the tested compounds act as inhibitors; in order to inhibit Alr, the compounds had to enter into the narrow well.

Confluentin (**1a**) was the best scored compound against TopoIV with a predicted Ki of 0.26 μM (Table 2). This compound interacts with TopoIV by forming hydrogen bonds with ARG-117 and TYR-180 (Figure 2F). The same compound also had the best score for DHFR protein with a predicted score of 0.44 μM (Table 2). This compound occupies the same area of Q27, a co-crystallized ligand. Furthermore, both compounds shared some hydrogen bonds with residues LEU-6 and PHE-93 (Figure 2G).

For DHPS protein, neogrifolin (1c) was the best compound scored (Table 2). DHPS catalyzes the condensation of PABA with 6-hydroxymethyl-7,8-dihydropterin-pyrophosphate (DHPP) to form 7,8-dihydropteroate [13]. Neogrifolin does not occupy the same area of the co-crystallized ligand PMM, an analog of DHPP. However, the compound occupied the region of *p*-hydroxybenzoic acid, which is an analog of PABA co-crystallized with DHPS from *Bacillus anthracis* (3TYB).

Neogrifolin and *p*-hydroxybenzoic acid were superimposed to the protein structure used in the present study (2VEG). The place where neogrifolin, PABA and *p*-hydroxybenzoic acid interact with DHPS is assumed to be the same places for sulphonamides or sulpha drugs interact, which are PABA analogues and act as alternate substrates for DHPS [14].

## 3. Experimental

### 3.1. Proteins and Natural Compounds Structure Preparation

The protein crystal structures were obtained from the Protein Data Bank (PDB): PBP1a with PDB entry 3UDI [15], Alr with PDB entry 2RJG [16], Ddl with PDB entry 2ZDQ (not published), IARS with PDB entry 1JZQ [17], DNA gyrase with PDB entry 3TTZ [18], TopoIV with PDB entry 3RAE (not published), DHPS with PDB entry 2VEG [19] and DHFR with PDB entry 3SRW [20].

The co-crystallized ligands: penicillin G (PNM) for PBP1a, ATP for Ddl, *N*-[isoleucinyl]-*N*'-[adenosyl]-diaminosufone (ILA) for IARS, 2-[(3*S*,4*R*)-4-{[(3,4-dichloro-5-methyl-1*H*-pyrrol-2-yl)carbonyl]amino}-3-fluoropiperidin-1-yl]-1,3-thiazole-5-carboxylic acid (07N) for DNA gyrase, (3*S*)-9-fluoro-3-methyl-10-(4-methylpiperazin-1-yl)-7-oxo-2,3-dihydro-7*H*-[1,4]oxazino[2,3,4-ij]-quinoline-6-carboxylic acid (LFX) for TopoIV, pterin-6-yl-methyl-monophosphate (PMM) for DHPS and 7-(2-ethoxynaphthalen-1-yl)-6-methylquinazoline-2,4-diamine (Q27) for DHFR, were separated from the corresponding protein and used to perform the docking validation. All crystallized water molecules were removed from all the structures except in three cases: 128 from 3SRW; A-2049 from 2VEG and A-2 from 3TTZ. These water molecules were found to be essential for a correct docking pose, since they improved docking pose for the tested compounds in the positive validation, decreasing RMSD. AutoDockTools1.5.2 (ADT) [21] was then used to assign polar hydrogens, add Gasteiger charges and save all protein structures in PDB file format. AutoGrid4 [22] was subsequently used to create all atom affinity grid maps, centred for each structure in the co-crystallized ligands, and with the necessary dimensions to encompass the ligand binding site.

The compound dataset used included 34 molecules reported in wild mushrooms that have been related to their antimicrobial activity [11,12]. ACD/ChemSketch Freeware 12.0 software was used to design 2D structure for all compounds and OpenBabel [23] was then used to perform 2D to 3D structure conversion. ADT was used to merge nonpolar hydrogens, add Gasteiger charges and set up rotatable bonds through AutoTors.

### 3.2. Molecular Docking

AutoDock4 (version 4.2) [22] with the Lamarckian genetic algorithm was used to perform the docking studies. Docking parameters selected for AutoDock4 runs were as follows: 50 docking runs, population size of 200, random starting position and conformation, translation step ranges of 2.0 Å, mutation rate of 0.02, crossover rate of 0.8, local search rate of 0.06, and 10 million energy evaluations. Docked conformations were clustered using a tolerance of 2.0 Å RMSD. The molecular docking experiments were performed on a dedicated cluster of 64 Core AMD 2.0 GHz, running on CentOS and using MOLA, a custom designed software for virtual screening using AutoDock [24]. All figures with structure representations were produced using PyMOL [25].

### 3.3. Docking and Score Validation

For docking validation, the molecular docking methodology described above was used to dock co-crystallized ligands PNM, ATP, ILA, 07N, LFX, PMM and Q27 against the respective structures. The result of the top conformation predicted by AutoDock4 was then compared to experimental co-crystallized binding pose and RMSD values were calculated.

For score validation, docking pose with the best score of ILA, 07N, PMM and Q27 were subject to a rescoring step using Xscore [26]. AutoDock4 and Xscore scores were then compared to experimental inhibition values, and ρ (Spearman rank correlation) and r (Pearson correlation coefficient) were calculated. Since the score values belong to different scales, both predicted and experimental Ki values were converted to pKi values before r calculation. 

### 3.4. Virtual Screening

The docking methodology described above was applied to dock the 34 compounds used as dataset against structures of 3UDI, 2RJG, 2ZDQ, 1JZQ, 3TTZ, 3RAE, 2VEG and 3SRW. For each compound, the docking pose with the best score was subject to a rescoring step using Xscore. Also, PyMOL was used to manually analyse the docking poses, in order to verify the pose occupied in the active site of each protein.

## 4. Conclusions

The data presented herein highlighted some conclusions regarding the affinity of different mushroom compounds to protein targets related to antibacterial action. Some compounds did not exhibit affinity for the target proteins considered, so possibly they use other target structures to exert their activity. However, several compounds (namely, the enokipodins, ganomycins and austrocortiluteins) indicated that main mechanism of their action is the inhibition of cell wall synthesis, being Alr and Ddl probable protein targets. Nevertheless, mushroom compounds might interact with other targets involving different mechanisms of action. Furthermore, docking studies were performed for only 34 selected compounds toward protein targets from specific microorganisms. However, some relevant affinities of compounds were observed in the docking study, which could indicate possible mechanisms.
